# Spatiotemporal Phytochrome Signaling during Photomorphogenesis: From Physiology to Molecular Mechanisms and Back

**DOI:** 10.3389/fpls.2016.00480

**Published:** 2016-04-11

**Authors:** Beronda L. Montgomery

**Affiliations:** ^1^Department of Energy — Plant Research Laboratory, Michigan State UniversityEast Lansing, MI, USA; ^2^Department of Biochemistry and Molecular Biology, Michigan State UniversityEast Lansing, MI, USA

**Keywords:** interorgan signaling, light signaling, organ-specific responses, photomorphogenesis, phytochrome, spatiotemporal responses, tissue-specific responses

## Abstract

Light exposure results in distinct responses in specific seedling tissues during photomorphogenesis. Light promotes growth of cotyledons and leaves, as well as development and elongation of roots, whereas light inhibits elongation of hypocotyls. For distinct plant responses such as shade avoidance, far-red light or shifts in spectral light quality similarly have disparate impacts on distinct plant tissues, resulting in elongation of stems or petioles and a reduction in growth of leaf blades for many species. The physiological bases of such tissue- and organ-specific light responses were initially studied using localized irradiation of specific tissues and organs, or irradiation of dissected plant parts. These historical approaches were used to identify spatial-specific pools of photoreceptors responsible for regulating local, i.e., tissue- or organ-specific, or distal, i.e., interorgan, plant responses. The red/far-red responsive phytochromes have been the most widely studied among photoreceptors in this regard. Whereas, the spatial localization of photoreceptors regulating many tissue- or organ-specific light responses were identified, the underlying signaling networks responsible for mediating the observed responses have not been well defined. Recent approaches used to investigate the molecular bases of spatiotemporal light responses include selective irradiation of plants harboring mutations in specific photoreceptors, tissue-specific expression of photoreceptors, primarily in photoreceptor mutant backgrounds, or tissue-specific biochemical ablation of photoreceptor accumulation. Progressive integration of such approaches for regulating the availability of localized pools of phytochromes with the use of transcriptomic or proteomic analyses for assessing the genes or proteins which these spatially discrete pools of phytochrome regulate is yielding emergent insight into the molecular bases of spatiotemporal phytochrome signaling pathways responsible for regulating spatiotemporal light responses of which we have been aware of at the physiological level for decades. Here, I discuss historical and emerging approaches to elucidating spatiotemporal signaling mediated by phytochromes during photomorphogenesis.

## Introduction

Plants exhibit developmental plasticity or an adaptive ability to alter growth and development in response to external cues. Among important environmental signals, light greatly impacts plant growth and development, productivity, and survival. Major photoreceptor families responsible for light perception and signaling in plants include the widely studied red (R)/far-red (FR) reversible phytochromes, blue (B)/ultraviolet-A (UV-A) responsive cryptochromes and phototropins, and ultraviolet-B (UV-B) absorbing photoreceptors, including UVR8 (Josse et al., 2008; Franklin and Quail, 2010; Kami et al., 2010; Rizzini et al., 2011; Galvão and Fankhauser, 2015; Li and Mathews, 2016). The integrated signaling driven by these photoreceptors results in the regulation of numerous light-dependent growth and developmental responses, including seed germination, transition from skotomorphogenesis (i.e., dark-mediated seedling morphology) to photomorphogenesis (i.e., light-dependent morphology and growth), leaf development, chloroplast differentiation and development, and other processes throughout the life cycle, such as flowering and ultimately senescence.

**FIGURE 1:**
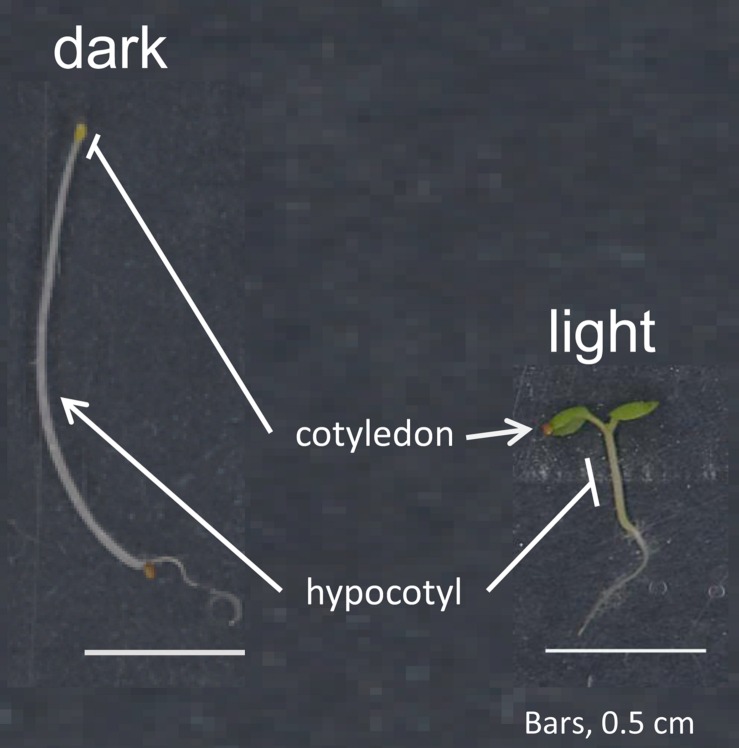
**Photomorphogenesis in seedlings.** Shown are *Arabidopsis thaliana* seedlings grown in either complete darkness (left) or in continuous white light (right). Light exposure of seedlings results in inhibition of growth in the hypocotyl, whereas light promotes expansion and development of cotyledons (and later true leaves) as well as root development, including lateral root formation and root hair initiation.

Light has distinct effects on different tissues during the process of photomorphogenesis and throughout various stages of the plant life cycle. During photomorphogenesis, light inhibits growth in the hypocotyl, but promotes growth and development in cotyledons and emerging true leaves, as well as in roots (**Figure 1**). Such divergent responses in distinct tissues could be maintained through having distinct pools of photoreceptors regulating the promotion of growth in cotyledons or roots, and distinct photoreceptors inhibiting hypocotyl elongation. Indeed, phytochromes and cryptochromes, which have critical roles in photomorphogenesis, accumulate at different levels and patterns in distinct tissues and due to developmental cues (Adam et al., 1994; Somers and Quail, 1995a,b; Goosey et al., 1997; Nagatani, 1997; Tóth et al., 2001; Sharrock and Clack, 2002). However, these photoreceptors also exhibit significant overlap in their patterns of expression, which do not fully support a role for spatially distinct photoreceptors in the control of divergent light-dependent growth responses in different tissues (Tóth et al., 2001). Thus, the distinct impacts of light on promoting growth in some tissues and inhibiting expansion in others is likely due to distinct signaling cascades downstream of the activated photoreceptors in distinct tissues. Throughout the last decade or so, the history of and advances in understanding tissue- and organ-specific light signaling, or spatiotemporal light signaling, during plant development and how these responses are coordinated have been discussed (Jiao et al., 2007; Bou-Torrent et al., 2008; Endo and Nagatani, 2008; Josse et al., 2008; Montgomery, 2008; Endo et al., 2016). Although initially identified and studied at the physiological level, insight into the molecular bases of spatiotemporal phytochrome responses and distinct players in the regulation of tissue- and organ-specific light-dependent responses is emerging.

## Historical and General Approaches to Investigating Spatiotemporal Phytochrome Responses

A number of different experimental approaches have been used to investigate the roles of photoreceptors in regulating tissue- or organ-specific light responses in plants. Historically, localized irradiation or microbeam irradiation was used to activate a small, and spatially limited, pool of photoreceptors and subsequently the impact of localized irradiation on responses in local or distal tissues was assessed. Tissue dissection and irradiation also have been used to identify spatial-specific light responses in plant tissues. Here, I focus primarily on investigations into the roles of phytochromes in spatiotemporal light responses in plants.

### Microbeam Irradiation

Microbeam or localized tissue irradiations have long been used to explore tissue-specific photoreceptor regulation of distinct aspects of plant growth and development. These experiments largely provided physiological evidence for organ- and tissue-specific photoreceptor responses, as well as insight into interorgan responses. Localized irradiation studies indicated distinct responses in nearly every tissue of seedlings, including cotyledons, hypocotyls and roots. Early studies uncovered light-dependent organ-autonomous responses and interorgan coordination of plant growth and development. Cotyledon-specific photoreceptors regulate interorgan signals controlling hypocotyl elongation and hook opening (Klein et al., 1956; De Greef and Caubergs, 1972a; Black and Shuttleworth, 1974; Caubergs and De Greef, 1975; Powell and Morgan, 1980). Such a response would be critically important in natural contexts for positioning leaves in optimal light environments. Cotyledons and leaves in an optimal light context for promoting photosynthesis and limiting photoinhibition or light-associated damage would be primed to send a strong signal to hypocotyls or stems to inhibit their elongation to maintain a favorable position. Alternatively, when the photosynthetic organs are in a less than favorable environment, a signal may be propagated to stems to initiate elongation in order to allow foraging for more optimal positioning in the photoenvironment.

Local and interorgan signals also control distinct aspects of leaf development, including leaf expansion (De Greef and Caubergs, 1972b) and plastid development and greening (De Greef et al., 1971; De Greef and Verbelen, 1972). As a part of leaf development, local and long distance phytochrome-dependent signals contribute to *CAB* gene expression (Bischoff et al., 1997). Similarly, anthocyanin accumulation in leaves was determined to be regulated by local and intertissue signals using localized irradiation (Nick et al., 1993). Interorgan signals from shoots also can impact root development (Tóth et al., 2001; Correll and Kiss, 2005; Salisbury et al., 2007), among other aspects of deetiolation in several plant species (Oelze-Karow and Mohr, 1974; De Greef et al., 1975; Lecharny, 1979; Mandoli and Briggs, 1982; Tanaka et al., 2002). Again, such responses are critical for integrative development of separate organs of the plant. In addition to shoot-originated interorgan signals impacting roots, identified local roles for photoreceptors in roots also emerged from studies using localized irradiation of seedlings (Jaffe, 1970; Tepfer and Bonnett, 1972). Although a role for photoreceptors, such as phytochromes, in roots that are generally growing below soil may be somewhat counterintuitive, phytochrome genes are expressed in roots (Tóth et al., 2001) and root-localized phytochrome holoproteins have similar light-activated dynamics as phytochromes in shoots (Chen et al., 2003). Root-localized phytochromes have documented roles in regulating primary and lateral root development in *Arabidopsis* (Reed et al., 1993; Salisbury et al., 2007).

Localized, or spot-light irradiation, also was used to determine the tissue specificity of additional responses in plants. One response investigated was phototropism, in which phytochromes have regulatory roles together with phototropins. Localization of phototropic control was distinct for monocots vs. dicots, with the topmost part of the hypocotyl being identified simultaneously as the local photoperception region and the light-responsive area where actual bending occurs in the dicot *Arabidopsis* (Yamamoto et al., 2014). Comparatively, the site of light perception for phototropism is separate and distinct from the site of coleoptile bending in monocots (Briggs, 1963; Iino and Briggs, 1984). Tissue-specific responses during shade avoidance include phytochrome-dependent elongation of petioles and reduced growth of the lamina. Localized irradiation has been used to investigate such tissue-specific shade avoidance responses in some plants (Von Wettberg and Schmitt, 2005).

### Tissue Dissection and Irradiation

Some very early studies investigating tissue-specific light responses were conducted with isolated plant parts such as fruits. Detached tomatoes were used to demonstrate organ-specific phytochrome responses in fruits that were correlated with phytochrome-dependent regulation of carotenoid synthesis and accumulation (Piringer and Heinze, 1954; Khudairi and Arboleda, 1971; Thomas and Jen, 1975; Alba et al., 2000). Such responses can occur through tissue-specific regulation of a phytochrome-dependent effector, such as the fruit-specific regulation of phytoene synthase activity by phytochromes (Schofield and Paliyath, 2005). Alternatively, in some cases the expression of the phytochrome genes themselves is regulated in a tissue-specific fashion such as in rice (Baba-Kasai et al., 2014), which can contribute to tissue-specific light responses.

Studies with irradiation of dissected tissues or irradiation of whole seedlings or plants with specific tissues or organs blocked from light exposure, e.g., aluminum foil-covered plant parts or shoots of soil-grown plants, also have been conducted to gain insight into the roles of localized pools of phytochromes. The use of foil to target light exposure to distinct tissues provided support for the finding that light absorption by cotyledons results in a strong ‘halt’ signal being propagated to hypocotyls to inhibit elongation (Black and Shuttleworth, 1974). However, dissected stems also appear capable of perceiving far-red light and initiating organ-specific growth (García-Martínez et al., 1987), which may be associated with repositioning of the photosynthetic leaves in whole plants in natural environments as described above. Selective covering of plant parts during irradiation and localized irradiation also were used to identify local versus interorgan signals from cotyledons and leaves as important for initiating internode elongation in response to far-red treatment in Sinapis alba (Casal and Smith, 1988a,b).

In addition to the localized irradiation studies with roots introduced above (see Microbeam Irradiation), more recent studies in which *Arabidopsis* roots were either obscured from or exposed to light indicated a role of root-localized phytochromes in organ autonomous signaling and development, as well as in coordinating root-hypocotyl ratios (Novák et al., 2015). Additionally, a recent study using plants with darkened roots compared to light-exposed roots implicated phytochrome-dependent photomorphogenesis and its establishment of photosynthesis as important for cotyledon-induced sucrose serving as a component of an interorgan signal transmitted to impact root development in response to light (Kircher and Schopfer, 2012).

## Integrating Molecular Approaches Into Understanding Spatiotemporal Light-Dependent Regulation of Plant Growth and Development

### Examination of Tissue-Specific Expression of Light-Regulated Genes

As results began to emerge from studies such as those described above with isolated tomato fruits and dissected plant parts, the underlying cause of some spatiotemporal light responses became clear as being associated with photoreceptor-dependent, tissue-specific gene expression (Schofield and Paliyath, 2005). To gain greater insight into light-dependent regulation of gene expression in specific tissues, transcriptomic approaches emerged as a valuable tool to assess spatiotemporal gene expression in distinct tissues. In such studies, global tissue-specific gene expression in response to light was investigated in *Arabidopsis* (Jiao et al., 2005; Ma et al., 2005) and rice (Jiao et al., 2005). In *Arabidopsis*, data emerged supporting distinct gene expression profiles in cotyledons, organs which expand in response to light, compared to hypocotyls, which exhibit an inhibition of growth when exposed to light (Jiao et al., 2005; Ma et al., 2005). Distinct light-dependent expression of genes in cotyledons and the shoot apical meristem also were reported (López-Juez et al., 2008). In additional analyses of tissue-specific light regulon data for cotyledons vs. hypocotyls from Ma et al. (2005), it was determined that the opposing growth responses in cotyledons and hypocotyls in response to light were not associated with a simple opposite regulation of a core set of genes in different organs (Josse et al., 2008). These analyses suggested that distinct gene networks downstream of the phytochromes caused disparate growth responses in different tissues (Josse et al., 2008). Tissue-specific expression of shade-induced genes also has been observed and the expression of many of the identified shade-responsive genes is phytochrome-dependent (Nito et al., 2015). Using a combination of localized irradiation and collection of specific tissues for transcriptomic studies, interorgan phytochrome signaling, e.g., cotyledon to apex, and organ-autonomous signaling were implicated in plant spatiotemporal shade responses (Nito et al., 2015). Transcriptomics-based analyses of red light-exposed roots also have been used to determine root-specific phytochrome regulons (Molas et al., 2006).

### Examination of Physiology and Development in Specific Tissues or Organs of Phytochrome Mutants

Analyses of the development of specific tissues or organs in plants which possess mutations or deletions in specific phytochromes genes or deletions in genes encoding the phytochrome chromophore biosynthesis enzymes, which are responsible for synthesis of the single chromophore used by all phytochromes (Muramoto et al., 1999; Kohchi et al., 2001; Emborg et al., 2006), have been used to understand tissue-specific phytochrome responses. Tissue- or organ-specific gene expression analyses also have been conducted with phytochrome mutants to identify effectors associated with spatiotemporal phytochrome responses. Such studies have led to genetic associations of phytochromes, or specific phytochrome family members, and phytochrome-dependent effectors with physiological responses previously documented at the physiological level by exposing plants to wavelengths of light used to preferentially activate phytochromes.

Interorgan phytochrome-dependent regulation of hypocotyl growth was reported using a combination of mutant analyses and tissue-specific gene expression in *Brassica rapa* (Procko et al., 2014). Additionally, roles for phytochromes and cryptochromes in regulating the contrasting growth responses of the petiole compared to the lamina in shade avoidance have been noted (Kozuka et al., 2005). Local or systemic roles for phytochromes in the regulation of elongation or phototropic responses of roots also have been reported based on analyses of *phy* mutants and chromophore biosynthesis mutants (De Simone et al., 2000a,b; Correll et al., 2003; Kiss et al., 2003; Correll and Kiss, 2005; Molas and Kiss, 2008; Costigan et al., 2011; Sindelar et al., 2014).

Some of these studies linked tissue-specific regulation of distinct downstream effectors to photoreceptor function in spatiotemporal light regulation. For example, hypocotyl-specific regulation of beta-tubulin TUB1 was associated with PhyA and PhyB in such an approach (Leu et al., 1995). Additionally, mesophyll-specific CUE1 was identified as a regulator of gene expression in a tissue-specific light response (Li et al., 1995). Expression of *ACT7* in hypocotyls is regulated by light in a tissue-specific manner in the transition from etiolated growth during skotomorphogenesis to deetiolated growth that occurs during photomorphogenesis (McDowell et al., 1996). Additionally, specific light-regulated genes have been shown to contribute to hypocotyl-localized phytochrome responses based on reverse genetic analyses (Khanna et al., 2006).

### Tissue-Specific Expression of Photoreceptors and Photoreceptor-Dependent Effectors

Tissue-specific expression of photoreceptors in wild-type or particularly in null mutant backgrounds emerged recently as a powerful tool to probe spatiotemporal pools of photoreceptors controlling distinct aspects of growth or development. Such an approach has been used successfully for both cryptochromes and phytochromes (Endo et al., 2005; Endo et al., 2007; Endo and Nagatani, 2008). Directed-overexpression of *PHYA* and localized plant irradiation were employed to probe tissue-specific roles of the phyA photoreceptor in tobacco (Rousseaux et al., 1997). These studies confirmed a role for leaf-localized phyA in some localized FR light-induced responses such as the regulation of chlorophyll content or specific leaf weight, as well as in regulating stem elongation through an interorgan-dependent signal (Rousseaux et al., 1997). Additionally, roles for tissue-specific phyB emerged from targeted gene expression studies, including evidence that mesophyll-specific phyB regulates an intertissue signal controlling flowering and hypocotyl elongation (Endo et al., 2005). Tissue-specific expression of phyB in a *phyB* null mutant background also showed that phyB in the mesophyll, phloem, or stomata all restore stomatal development, indicating that a phyB-dependent signal from leaves is functioning locally in the stomata or generates an intertissue, or systemic, response (Casson and Hetherington, 2014). A similar approach has been used to investigate tissue-specific roles of the B/UV-A cryptochrome 2 receptor in regulating flowering in *Arabidopsis* (Endo et al., 2007), as well roles for phototropin 2 in tissue-autonomous regulation of palisade cell development in leaves (Kozuka et al., 2011).

Following success with tissue-specific expression of photoreceptors in mutant backgrounds as an effective approach for identifying spatiotemporal pools of receptors controlling development, similar approaches for studying the photoreceptor-associated effectors have begun to emerge. Tissue-specific expression of *SPA1*, for example, has indicated that specific pools of SPA1 regulate distinct aspects of seedling de-etiolation, leaf development, or SPA-dependent regulation of flowering induction (Ranjan et al., 2011). Recent studies investigating the roles of phytochrome-interacting factor PIF7 in the spatially specific shade avoidance responses implicated PIF7 in tissue-specific growth responses during shade detection (de Wit et al., 2015). Also, root localized photoreceptors, including phytochromes, regulate root-specific effector SCAR during light-dependent regulation of root elongation and development (Dyachok et al., 2011).

### Tissue-Specific Ablation of Photoreceptors

Transgenic modulation of protein synthesis or accumulation has been proposed as a valuable method for investigating protein signaling networks *in planta* (Warnasooriya and Montgomery, 2011b). A transgenic approach targeting degradation of the tetrapyrrole phytochrome chromophore *in vivo* to regulate accumulation of phytochromes has been developed as a robust tool for investigating phytochrome functions (Lagarias et al., 1997; Montgomery et al., 1999, 2001). Given that phytochromes covalently attach a dedicated chromophore, as compared to blue light photoreceptors that use flavin-based chromophores that serve additional roles in cells, modulation of the phytochrome chromophore can be utilized specifically to regulate phytochrome synthesis and accumulation *in vivo*. Use of a phytochrome chromophore degrading tool in a tissue-specific fashion, then, allows probing spatiotemporal phytochrome responses. Tissue-specific induction of localized phytochrome deficiency has been used to provide molecular evidence for a role of cotyledon- or leaf-localized phytochromes in the regulation of hypocotyl elongation (Montgomery, 2009; Warnasooriya and Montgomery, 2009), which had previously been observed at the physiological level using localized irradiation of plants (discussed above in Section “Microbeam Irradiation”). Additionally, the regulation of organ-specific anthocyanin accumulation was identified through targeted phytochrome ablation (Warnasooriya et al., 2011). Such studies also led to insights into the roles of root-specific phytochromes and phytochrome-driven shoot–root interactions in the light-dependent regulation of root development and elongation (Costigan et al., 2011; Warnasooriya and Montgomery, 2011a; Hopkins and Kiss, 2012). A role for shoot meristem-specific phytochromes in the photoperiod-dependent regulation of rosette leaf size and number also was reported based on targeted inactivation of the phytochrome chromophore (Warnasooriya and Montgomery, 2009). This latter finding corresponds to the identification of a shoot apex-specific set of light-regulated genes in prior studies (López-Juez et al., 2008).

The isolation of lines with distinct patterns of phytochrome deficiency served as genetic resources for use in proteomic (Oh and Montgomery, 2011) or transcriptomic studies (Oh et al., 2013) to identify specific factors functioning downstream of the phytochromes that are involved in spatiotemporal phytochrome responses, such as the interorgan cotyledon-dependent regulation of hypocotyl elongation. Microarray analyses of lines with induced deficiencies in mesophyll phytochromes resulted in the identification of specific factors whose expression was induced in cotyledons, but which impacted hypocotyl elongation (Oh et al., 2013). Furthermore, specific factors were identified that were involved in phytochrome-dependent anterograde signaling between nucleus and chloroplasts and aspects of deetiolation (Oh and Montgomery, 2013, 2014). Although a role for light in coordinating expression of nuclear and chloroplast genomes had been recognized previously as important for seedling establishment (Hoober et al., 2007; Waters et al., 2008), the specific photoreceptors and their tissue localization or site of action emerged from an ability to manipulate phytochrome levels in a spatial-specific fashion (Oh and Montgomery, 2013, 2014). Proteomic studies of lines with localized phytochrome chromophore depletion identified several beta-gluosidase proteins as targets of local and systemic repression by mesophyll-specific phytochrome signaling (Oh and Montgomery, 2011). For additional future advances, combining tissue dissection or organ-specific analyses with tissue-specific ablation of phytochromes has the potential to lead to the identification of additional effectors involved in very specific aspects of spatiotemporal phytochrome-dependent responses (Warnasooriya and Montgomery, 2010).

## Perspective

Newly emerging tools and genetic advances are allowing innovative experiments to be conducted that revisit the long known tissue- and organ-specific, as well as long-distance interorgan, physiological responses to light in plants. To date, such studies indicate that phytochromes initiate distinct signaling cascades in different tissues which result in the divergent responses seen to light. These spatiotemporal phytochrome responses are central to coordinated plant growth, development and metabolism, yet we are truly at the forefront of understanding the structure and coordination of the signaling networks downstream of the photoreceptors that impact distinct light-dependent growth responses. An ongoing focus on applying cutting-edge techniques to address the roles of localized pools of phytochromes in regulating and coordinating cell, tissue, and organ autonomous photoresponses, as well as in initiating interorgan signaling required for whole plant responses will continue to expand our knowledge of the mechanisms important for mediating distinct aspects of plant growth and development. Furthermore, follow up studies on the phytochrome-dependent factors and signals which comprise the spatiotemporal phytochrome signaling networks are anticipated to lead to greater understanding of the molecular basis of these recognized spatiotemporal phytochrome-dependent responses. Such advances in knowledge are essential for developing key tools that will allow targeted control of such responses of agronomic or biotechnological value.

## Author Contribution

The author confirms being the sole contributor of this work and approved it for publication.

## Conflict of Interest Statement

The author declares that the research was conducted in the absence of any commercial or financial relationships that could be construed as a potential conflict of interest.
